# Supplementary figures and images for: The soil microbiomics of intact, degraded and partially-restored semi-arid succulent thicket (Albany Subtropical Thicket)
Source: PeerJ. 2021 Oct 6;9:e12176. doi: 10.7717/peerj.12176 (PMC8501999; doi:10.7717/peerj.12176)

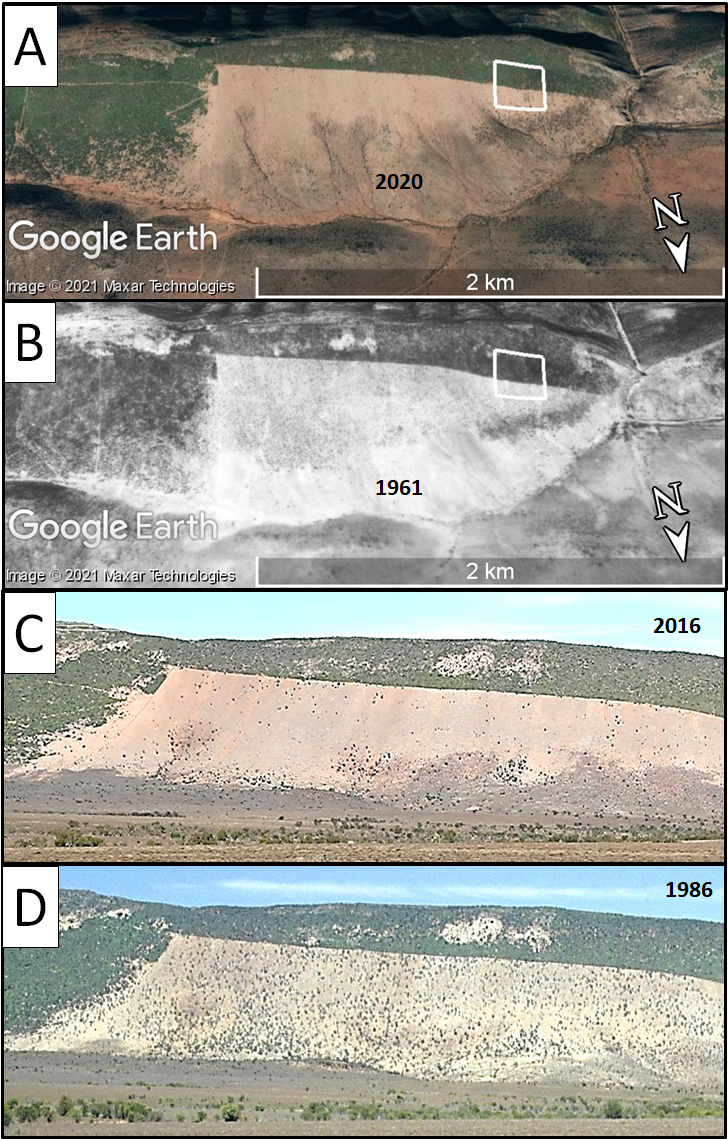

Supplement: Supplemental Information 1 — Aerial images show that this degraded state has been in effect for at least 60 years: (A) satellite image from 2020 (Map data: ©2021 Google Earth, Maxar Technologies), (B) aerial photo taken in 1961 by the South African Chief Directorate of National Geo-spatial information (Reproduced under Government’s Printer Authorisation [Authorisation No. 11851 dated 08 September 2021]). Ground-based repeat photography demonstrates the loss of the majority of remaining trees since 1986: photos taken in (C) 2016 and (D) 1986 (Photo credit: MT Hoffman). Note that, in (C) and (D), the area in this study is not in the area photographed (it is off to the right). [file peerj-09-12176-s001.png]
