# Supplementary material for: The soil microbiomics of intact, degraded and partially-restored semi-arid succulent thicket (Albany Subtropical Thicket)
Source: PeerJ. 2021 Oct 6;9:e12176. doi: 10.7717/peerj.12176 (PMC8501999; doi:10.7717/peerj.12176)
Supplement: Supplemental Information 16 — Prevalence thresholds are given both as a percentage (%) and as the equivalent number of sites (n) in the intact and degraded zones. The thresholds and resultant ASV counts analysed in this study are shown in bold [file peerj-09-12176-s016.docx]

|  |  |  | **Prevalence out of zone** | | | |  |  |
| --- | --- | --- | --- | --- | --- | --- | --- | --- |
|  | **%** |  | 0 | 5 | **10** | 20 |  |  |
|  |  | **n** | 0 | 1 | **2** | 3 |  |  |
| **Prevalence within zone** | 100 | 15 | 0 | 0 | 0 | 1 | **Intact** | **Zone** |
|  | **95** | **14** | 2 | 2 | **2** | 5 |  |  |
|  | 85 | 13 | 2 | 2 | 2 | 5 |  |  |
|  | 80 | 12 | 3 | 3 | 3 | 6 |  |  |
|  | 100 | 15 | 1 | 1 | 1 | 1 | **Degraded** |  |
|  | **95** | **14** | 2 | 2 | **2** | 2 |  |  |
|  | 85 | 13 | 3 | 3 | 3 | 3 |  |  |
|  | 80 | 12 | 3 | 3 | 3 | 4 |  |  |
